# Molecular clonality analysis of esophageal adenocarcinoma by multiregion sequencing of tumor samples

**DOI:** 10.1186/s13104-017-2456-5

**Published:** 2017-04-04

**Authors:** Anna M. J. van Nistelrooij, Ronald van Marion, Linetta B. Koppert, Katharina Biermann, Manon C. W. Spaander, Hugo W. Tilanus, J. Jan B. van Lanschot, Bas P. L. Wijnhoven, Winand N. M. Dinjens

**Affiliations:** 1 Department of Pathology, Erasmus MC Cancer Institute, University Medical Center Rotterdam, P.O. Box 2040, 3000 CA Rotterdam, The Netherlands; 2 Department of Surgery, Erasmus MC Cancer Institute, University Medical Center Rotterdam, P.O. Box 2040, 3000 CA Rotterdam, The Netherlands; 3 Department of Gastroenterology & Hepatology, Erasmus MC Cancer Institute, University Medical Center Rotterdam, P.O. Box 2040, 3000 CA Rotterdam, The Netherlands

**Keywords:** Esophageal adenocarcinoma, Barrett’s esophagus, Intratumor heterogeneity, Clonality analysis

## Abstract

**Background:**

Intratumor heterogeneity has been demonstrated in several cancer types, following a model of branched evolution. It is unknown to which extent intratumor heterogeneity is applicable to esophageal adenocarcinoma. Therefore the aim of this study was to characterise intratumor heterogeneity in esophageal adenocarcinoma.

**Methods:**

Multiregional targeted sequencing of four commonly altered genes was performed on 19 tumor regions collected from five esophageal adenocarcinomas. Alterations were classified as homogeneous or heterogeneous based on mutational and loss of heterozygosity analysis.

**Results:**

Identical *TP53* mutations and homogeneously loss of heterozygosity of the *TP53* locus were identified in all separated tumor regions in each of five adenocarcinomas, and in the corresponding Barrett’s esophagus and tumor positive lymph node of one primary tumor. Loss of heterozygosity of the *P16* locus was homogeneous among all tumor regions in four adenocarcinomas, and an identical pattern of loss of heterozygosity was present in the Barrett’s esophagus. Loss of heterozygosity of the *SMAD4* and *APC* loci was observed in a heterogeneous pattern.

**Conclusions:**

Known driver alterations, such as *TP53* and *P16* are homogeneously present within each adenocarcinoma, and therefore occur early during carcinogenesis and subsequently clonally expand throughout the entire tumor. However, loss of heterozygosity of the *SMAD4* and *APC* loci shows a heterogeneous pattern, indicating intratumor heterogeneity of esophageal adenocarcinoma.

## Background

The incidence of esophageal adenocarcinoma (EAC) has been rising rapidly in Western countries over the last decades [1–3]. The major risk factor for EAC is Barrett’s esophagus (BE) [4], a premalignant condition, in which the normal squamous epithelium of the distal esophagus has been replaced by columnar epithelium, including goblet cells. The risk of developing EAC from BE is estimated at 0.12–0.5% per year and follows a multimorphological sequence, in which metaplasia evolves into low-grade dysplasia (LGD), high-grade dysplasia (HGD) and ultimately into EAC [5–7].

Previous results from whole-exome/genome sequencing of EACs demonstrated that these tumors bear a broad mutational spectrum; genes frequently altered are e.g. *TP53*, *P16*, *SMAD4* and *APC* [8–10]. Alterations in the *TP53* gene occur in the majority of EAC cases, however, only few other somatic alterations are shared between EACs, representing substantial intertumor heterogeneity. In addition, emerging evidence suggests that even an extensive molecular variation is present within individual tumors, termed intratumor heterogeneity [11–13].

With the use of Next-generation sequencing, intratumor heterogeneity has been demonstrated in several cancer types and even a model of branched evolution leading to intratumor heterogeneity has been advocated [12, 14]. This model implicates that a single biopsy may not represent the total mutational burden of a tumor, which can explain the heterogeneous mutational spectrum described in EACs [8]. Conceivably, intratumor heterogeneity may lead to an underestimation of the mutational spectrum of a tumor using a single biopsy procedure, which might clarify the difficulties in finding and validating clinically valuable oncological biomarkers.

To date it is unknown to which extent this model of branched evolution for intratumor heterogeneity is applicable to EAC. Therefore, the aim of this study was to characterize intratumor heterogeneity in EAC, by multiregional targeted sequencing on a total of 19 tumor regions collected from five EAC patients, including adjacent BE in one of them, who underwent primary surgical resection with curative intent. To evaluate intratumor heterogeneity, DNA alterations were classified as homogeneous, i.e. present in all regions of the tumor, or heterogeneous defined as present in some regions or in only one region of the tumor.

## Methods

### Patients and specimens

Resection specimens of distal esophageal or gastro-esophageal junction adenocarcinomas were obtained from five patients treated between December 2001 and April 2002 at the Department of Surgery, Erasmus MC Cancer Institute, University Medical Center Rotterdam, The Netherlands. All patients underwent a transhiatal esophagectomy, and none of them received neoadjuvant chemo- or radiation therapy, which is part of the current standard treatment of EAC [15].

### Tissue samples, DNA isolation and immunohistochemistry

Tissue samples were derived from non-malignant and malignant areas in the fresh resection specimens and used according to the Code of Proper Secondary Use of Human Tissue in the Netherlands, as established by the Dutch Federation of Medical Scientific Societies (http://www.federa.org). In addition the study was approved by The Medical Ethical Committee of the Erasmus MC, University Medical Center, Rotterdam (MEC-2016-067).

**Fig. 1:**
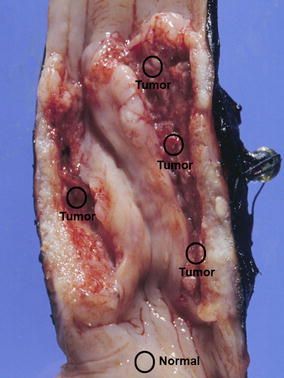
Macroscopically separated tumor regions of resection specimen

From each of the five EACs, multiple, macroscopically separated tumor regions were collected (Fig. 1). In addition, from one resection specimen, samples from an area of premalignant BE (HGD) and from a tumor positive lymph node (TPLN) were available for study. Tumor tissue areas composed of at least 50% neoplastic cells (confirmed by a GI-pathologist) were manually microdissected from 10 to 15 hematoxylin-stained Sects. (4 µm) of formalin-fixed paraffin-embedded tissue blocks. DNA was extracted using proteinase K and 5% Chelex 100 resin. TP53 immunohistochemistry was performed with the mouse monoclonal antibody Do-7 (Dako, Glostrup, Denmark), according to standard protocols.

### Ion torrent personal genome machine

Ion semiconductor sequencing was performed on the ion torrent personal genome machine (PGM) with a custom-made cancer panel on DNAs extracted from the macroscopically separated tumor regions according to the manufacturer’s protocols. In short, libraries were made using the Ion AmpliSeq Library Preparation Kit. A template was prepared using the Ion OneTouch Template Kit and sequencing was performed with the Ion Sequencing Kit v2.0 on an Ion 316 chip. Data were analysed with the Variant Caller v2.2.3-31149 (Life Technologies, Carlsbad, CA, USA). Variants were called when the position was covered at least 100 times. Sequences of all primers and probes are available on request.

### Mutation analysis

The custom-made cancer panel contained the genes: *APC*, *P16*, *SMAD4* and *TP53*. Of all genes the total coding regions and the exon–intron boundaries were covered. Nonsynonymous somatic point mutations, insertions and deletions that change the protein amino acid sequence and splice site alterations were selected. In addition, variants present in the ESP6500si or 1000genomes databases in ≥1% were excluded. Variants present in at least 30% of the called reads were considered reliable and were validated by Sanger sequencing according to standard protocols.

### Loss of heterozygosity analysis

To demonstrate loss of heterozygosity (LOH) of the genes of interest (*APC*, *P16*, *SMAD4*, *TP53)* amplicons for single nucleotide polymorphisms (SNPs, selected from dbSNP137) in these loci were added to the custom-made cancer panel. Per locus nine SNPs were selected, all with a minor allele frequency of ≥0.45 and located within the gene (three SNPs) and at positions ~300, ~600 and ~900 kb centromeric and telomeric of the gene. The SNPs were considered heterozygous if the percentage of the variant in the normal DNA was within the range of 40–60%. When at least one SNP was present heterozygous in the normal DNA it was considered informative for LOH analysis. Variants present in the normal DNA in <10% were considered homozygous reference and therefore not informative for LOH analysis, as well as variants present in >90%, which were considered homozygous variant.

For tumor DNAs, of which the normal DNA was considered heterozygous, all SNPs with variants <40 or >60% were denoted as indicative for LOH. A sample was evaluated as having locus LOH when ≥50% of the informative SNPs in that locus demonstrated LOH (variant in tumor DNA <40 or >60%) [16]. In addition, identified tumor suppressor gene mutations also supplied information about possible LOH (loss of the wild type allele) by the relative frequency of the mutant DNA sequence compared to the normal wild type sequence in the tumor samples.

### Intratumor heterogeneity analysis

Analysis of intratumor heterogeneity could be determined through mutation and LOH analysis. Homogeneity was signified by an identical mutation or the same pattern of LOH at a given gene identified in different tumor regions of the same tumor, whereas heterogeneity was signified by different mutations or various LOH patterns of a given gene in different regions of the same tumor.

## Results

Targeted sequencing with a custom-made cancer panel was performed on DNA isolated from 19 tumor regions derived from the resection specimen of five EAC patients; all male, with a median age at the time of diagnosis of 70 years (range 51–78 years). Two tumors were localised at the gastro-esophageal junction and three in the distal esophagus within the background of BE. Four tumors were moderately differentiated, and one tumor was poorly differentiated. All resection margins were free of tumor cells. Three patients had tumor positive lymph nodes, without distant metastasis. Patient and tumor characteristics are listed in Table 1.

**Table 1:** Patient and tumor characteristics

Patient	Tumor type	Differentiation grade	Resection margin	TNM stage^a^	Tumor regions
EAC1	GEJAC	Moderate	R0	pT3N1Mx	5
EAC2	GEJAC	Moderate/poor	R0	pT3N3Mx	4
EAC3	BAC	Moderate	R0	pT1N0Mx	3
EAC4	BAC	Moderate	R0	pT3N1M0	3
EAC5	BAC	Poor	R0	pT2N0Mx	4

*GEJAC* Gastro-esophageal junction adenocarcinoma

*BAC* Adenocarcinoma in Barrett’s esophagus

*R0* tumor free resection margin

^a^According to the classification of the American Joint Committee on Cancer (AJCC) Staging Manual 7th edition

From one resection specimen, a region of BE (HGD) and a TPLN were analysed with the custom-made cancer panel as well. Next-generation sequencing on the PGM and conventional Sanger sequencing revealed 21 mutations in the *TP53* gene, no mutations were identified in *APC*, *P16*, and *SMAD4*. Reliable data for the LOH analysis of *TP53*, *P16*, *SMAD4* and *APC* were obtained in 92, 92, 81 and 73% of the samples, respectively.

**Fig. 2:**
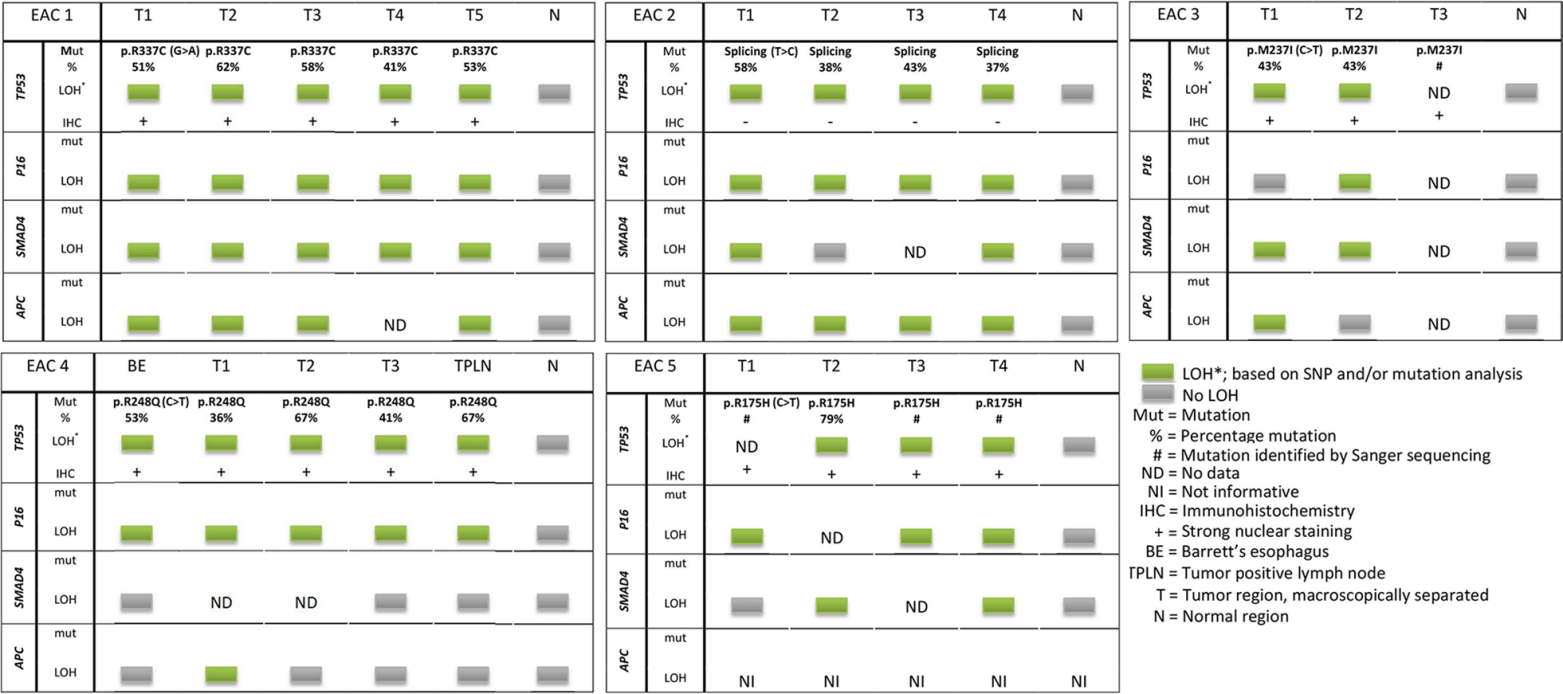
Overview of mutation and LOH analysis per tumor regions of five EACs

**Fig. 3:**
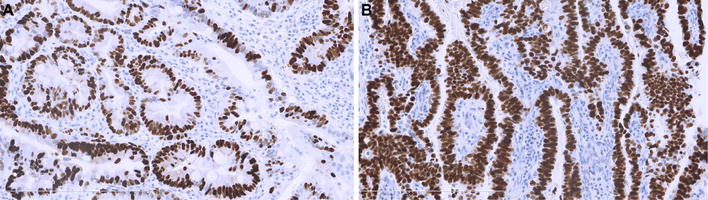
TP53 immunohistochemistry on Barrett’s high-grade dysplasia and invasive EAC tissue

All five EACs showed homogeneous *TP53* mutations: in each EAC the same *TP53* mutations (nonsynonymous somatic point mutations or splice site alterations) were identified in all investigated tumor regions, all mutations were previous described in esophageal cancer samples in the COSMIC database. In addition, the *TP53* mutation found in a primary EAC was also identified in the adjacent BE and TPLN samples. LOH of *TP53* occurred homogeneously in all informative regions of the EACs and in the paired EAC and BE samples with identical LOH patterns observed by SNP and/or mutation analysis (Fig. 2). TP53 immunohistochemistry showed homogeneous and strong nuclear expression in all tumor regions and the BE sample of the four patients with *TP53* somatic missense mutations (Fig. 3). The tumor cells of the four regions derived from the EAC with a *TP53* splice site mutation were all homogeneously negative for *TP53* expression (EAC2).

**Fig. 4:**
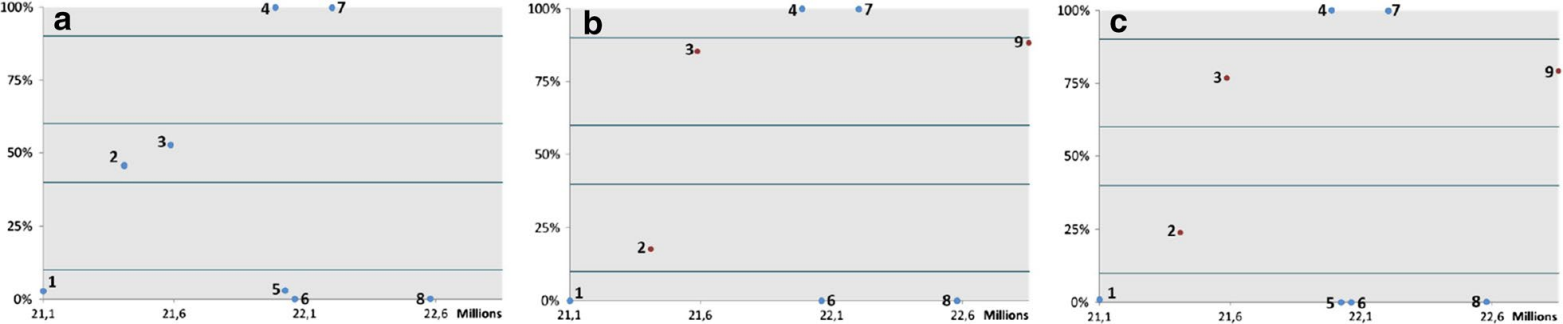
*P16* LOH SNP analysis of normal, Barrett’s high-grade dysplasia and concomitant EAC sample

LOH of *P16* occurred homogeneously in all regions of four informative tumors (EAC1, 2, 4, 5), and an identical LOH pattern of *P16* was observed in the paired tumor and BE samples (Fig. 4). EAC1 and EAC3 showed homogeneous LOH of *SMAD4* in all tumor regions, two tumors showed different subclones with LOH or without LOH of *SMAD4* (EAC2 and 5), while in EAC4 no LOH of *SMAD4* was identified. Homogeneous LOH of *APC* was observed in two tumors (EAC1 and 2), different subclones with and without LOH of *APC* were observed in EAC3 and 4, whereas one tumor (EAC5) was not informative for the *APC* locus (Fig. 2).

## Discussion

Recent data on Next-generation sequencing of several tumor DNAs were supportive for the model of branched tumor evolution leading to intratumor heterogeneity [11, 13]. This model describes a tumor as a tree structure, with the trunk representing early molecular alterations, which clonally expand and therefore are homogeneously present throughout the entire tumor, reflecting a process involved before and during tumor initiation and early development. The branches of the tree represent later molecular alterations, which as a result are only present in different subclones of the tumor, contributing to intratumor heterogeneity and shaping the genome during tumor maintenance and progression [12, 14]. The extent of this branched evolution model leading to intratumor heterogeneity in EACs is as yet unknown.

Therefore, the aim of the current study was to characterize intratumor heterogeneity in EACs, by performing multi-region, targeted sequencing on 19 tumor regions derived from five surgical resected EACs. Targeted sequencing of commonly altered genes; *TP53*, *P16*, *SMAD4* and *APC* was performed. This analysis revealed a clonal origin of *TP53* alterations: all five EACs where homogeneous with regard to *TP53* mutations and LOH of the *TP53* locus, in addition the same mutation and pattern of LOH in *TP53* was observed in a paired TPLN of one EAC. These results indicate that *TP53* mutation and LOH of the *TP53* locus are relatively early events in EAC tumorigenesis and clonally expand throughout the entire tumor. In concordance with this is the finding of the same *TP53* mutation, identical pattern of LOH, and comparable strong nuclear TP53 expression in the paired primary tumor and BE case.

Mutations in the *P16* gene were not observed in this study, however LOH of the *P16* locus was identified in all EACs. Four EACs had a homogeneous *P16* locus LOH pattern, while one EAC was heterogeneous for *P16* LOH. In addition, an identical pattern of *P16* locus LOH was present in the paired primary EAC and BE case, suggesting that LOH of *P16* is an early alteration, clonally expanding throughout the tumor. No mutations were identified in *SMAD4* and *APC*, but LOH of these genes was observed in a heterogeneous pattern within the EACs. Some EACs showed homogeneous LOH of *SMAD4* and/or *APC*, one EAC showed no LOH of *SMAD4* at all, while the remaining EACs showed different subclones: some with LOH of *SMAD4* and/or *APC* and others without LOH of these loci. In addition, no LOH of *SMAD4* and *APC* was found in the BE sample, indicating a late occurrence of these alterations, reflecting intratumor heterogeneity of EACs.

Taken together, the results of the current study suggest that both homogeneous and heterogeneous intratumoral molecular alterations are present in EACs. A homogeneously present *TP53* mutation and LOH of the *TP53* locus as well as LOH of the *P16* locus in the primary tumor were also found in the adjacent BE sample, indicating that the earliest molecular alterations can already be present in the premalignant lesion (BE) and from there on clonally expand throughout the entire tumor. Temporarily, no multiregion sequencing of EACs was performed before. Alterations in *TP53* were described previously in the sequential of BE (HGD) and EAC, and in addition were found to be present in a major clone, which also indicates clonal expansion of *TP53* as an early alteration [17].

The presence of alterations in BE is in accordance with previous studies, showing that multiple BE crypts contain different clones with alterations competing with each other [18, 19], of which one progenitor clone with a selective growth advantage will expand clonally and will create a field in which other (pre)malignant alterations might arise [19]. Several studies on *P16* have reported clonal expansion of *P16* alterations in all regions of BE segments, and it has been suggested that expansion of *TP53* alterations occurs only in a background of *P16* altered clones [20]. However, others described progenitor clones containing *TP53* alterations alone or in combination with *P16* alterations present at many levels of BE segments [21]. Leedham et al. [18] concluded that BE was genetically heterogeneous, however they observed identical *TP53* mutations in multiple BE crypts, indicating widespread and far-reaching clonal expansion as a consequence of the strong selective advantage that absence of *TP53* function supposedly provide. Recently, a study on cytochrome *c* oxidase (CCO) deficient cells in BE confirmed the concept of clonal expansion of BE, probably by fission of BE glands [22].

Conceivably, intratumor heterogeneity can cause tumor sample bias using single biopsy approaches, since it may underestimate the mutation spectrum of a tumor. This could contribute to difficulties in identifying and validating biomarkers, which are desirable to identify BE patients with a high risk for neoplastic progression. Furthermore, intratumor heterogeneity may contribute to therapy resistance: if the actionable target of treatment is only present in a subclone of the tumor, than targeting this genetic alteration may not have an impact on the entire tumor. This concept might explain the diversity in responders and non-responders after neoadjuvant chemoradiotherapy in EACs [23].

## Conclusions

Recent studies on EAC reveal extensive intertumor heterogeneity concerning molecular alterations with different studies showing different recurrently mutated genes. Therefore, the genes found frequently mutated in several studies were investigated in the current study [8–10, 17]. Even though the sample size is small, and targeted sequencing of only commonly mutated genes was performed, the current study provides evidence that although intratumor heterogeneity is present in EACs, known driver alterations in *TP53* and *P16* were homogeneously present in all five primary EACs, indicating clonal cellular expansion. Studies with larger cohorts and extensive genome sequencing are needed to fully characterize intratumor heterogeneity in EACs and in addition, to understand the impact of intratumor heterogeneity on current clinical outcome of both BE and EAC patients.
